# Calcium extraction from catfish bone powder optimized by response surface methodology for inducing alginate bead

**DOI:** 10.1016/j.heliyon.2024.e30266

**Published:** 2024-04-26

**Authors:** Manop Sriuttha, Nachayut Chanshotikul, B. Hemung

**Affiliations:** School of Applied Science, Faculty of Interdisciplinary Studies, Khon Kaen University, Nong Khai Campus, Nong Khai, 43000, Thailand

**Keywords:** Catfish bone powder, Microwave-assisted extraction, RSM, Calcium extraction, Alginate bead

## Abstract

Catfish bone powder (CBP), prepared from catfish head, was a good source of natural minerals, particularly calcium (Ca) and phosphorous (P). The Ca content were higher than P when analyzed by either chemical or SEM in EDS mode. These elements were found in the crystal form of hydroxyapatite (HA) complex with protein, as assessed by spectroscopic techniques, including XRD and FT-IR. Thus, low Ca solubility was thus observed at 0.03 ± 0.0038 % and digestion of complex HA is required for more liberation of Ca. Citric acid was therefore applied for Ca extraction using a microwave-assisted method. The conditions were optimized using response surface methodology with central composite design to evaluate the effect of extraction time, microwave power, citric acid concentration, and CBP weight. Based on a linear prediction model, the optimum condition to extract Ca from 0.4 g of CBP was at 0.1 M citric acid using microwave power of 275 W for 60 s. This condition provided a Ca content of 1.11 mg/mL in solution, which extract calcium about 27.75 % from original CBP weight. This Ca concentration was enough to induce alginate drops (1.0 %) to form hydro-beads, indicating its functional property in food system. These results provided the promising method to extract natural calcium from fishery by-products for further creating innovative food products.

## Highlights

• Catfish head could be a good source of natural calcium for food applications.
• Extraction of soluble calcium could be performed by microwave assisted extraction.
• Optimum condition for calcium extraction can be obtained by RSM methodology.
• Alginate bead could be induced by calcium ion extracted at the optimum condition.

## Graphical abstract

**Figure:**
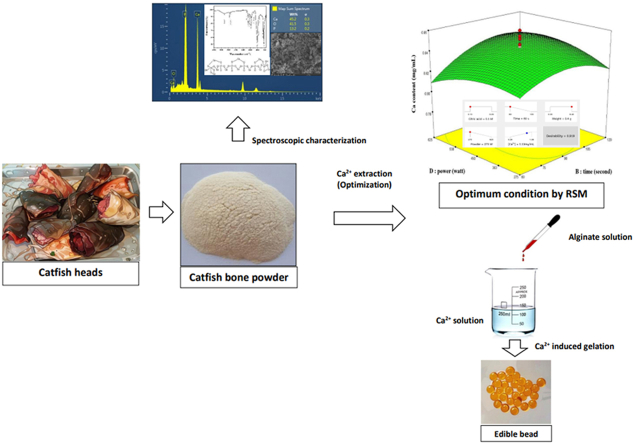

## 1 Introduction

Catfish (*Clarias macrocephalus*) production is one of economic aquacultures and this fish is widely grown in several wetlands throughout Thailand. Its annual production is about 99,344 MT which accounted for revenues of 145,577,000 US Dollars [1]. It is consumed either fresh or processed into other products. Initially, catfish must be eviscerated and the undesirable parts trimmed, *e.g.*, the head and gut. This adds waste management costs to the process, especially due to the fish heads. However, this waste can become a low-cost raw material for subsequent processing, since it contains bones that are a good source of natural minerals.

The bones of fish comprise approximately 10–15 % of their total body weight, including those in the body and appendages [2]. They consist of an organic extracellular matrix covered with hydroxyapatite [Ca_5_(PO_4_)_3_OH], HA. The calcium (Ca) content is as high as 13.5–14.7 % on a dry matter basis [3]. It is reported that bones from small fish such as anchovies and lizardfish can be consumed directly or processed into ready-to-eat foods rich in calcium [4]. However, Ca in the form of HA is not suitable for absorption into the human body [5]. Utilization of bone from large fish must be processed by removing protein and fat using alkali and an organic solvent, respectively. Thereafter, enzymatic hydrolysis prior crushing into powder is necessary. Yin et al. [6] reported that the particle size of fish bone powder is important for producing desirable functional properties. They determined that fish bone at nanoscale particle sizes provided better texture than those at a microscale. This is because small particles facilitate Ca liberation, resulting in activation of fish transglutaminase to catalyze protein cross-linking. Thus, very fine grinding is needed to produce nano-scale fish bone particle sizes and might be limited in some applications [7].

Extraction of Ca from fish bones was successfully done using several acid solutions [8]. Dewi [9] extracted nano-calcium from tilapia bone using hydrochloric and acetic acids under optimum conditions (0.3 N for 60 min). Extracting calcium using hydrochloric acid provided high yield and solubility but application of organic acids is highly absorbable calcium materials, as well as a good acid fortifier in food application. Extraction of Ca from shank bones by acetic and citric acid has been reported [10]. Although acid extraction of Ca from fish bone using classical methods is possible, the time required to complete such a process might not be practical.

Microwave-assisted extraction has been introduced to improve extraction efficiency of several compounds [11]. It offers several benefits, *e.g.*, rapid heating, shorter time, and more efficient energy transmission. The technique was used to reduce reaction time for calcium phosphate extraction from eggshells [12]. Replacement of the conventional extraction by a microwave assisted extraction enabled less solvent consumption with higher extraction from the calcium sennosides of senna leaves [13].

To determine the optimal conditions, several factors affecting extractability should be considered, *e.g.*, extraction time, microwave power, solute: solvent ratio and acid concentration. However, evaluation of more than two factors at the same time might be limited due to the simple experimental analysis used, complete randomized design. Response surface methodology (RSM) is applied to determine the best conditions at the lowest overall cost. It was applied to optimize the extraction conditions of gelation from catfish bones [14]. Optimization to obtain the highest calcium content in catfish bone flour has been successfully done using RSM [15]. Application of a microwave assisted acid extraction technique would likely be a promising approach to extract Ca, but it has not been reported for fish bones. Therefore, the objective of this study was to optimize the conditions for Ca extraction from bone powder prepared from catfish heads using a microwave assisted digestion employing citric acid as a solvent. Additionally, the capability of soluble Ca extracted at the optimal conditions to induce gelation of an alginate solution was evaluated.

## 2 Materials and methods

### 2.1 Catfish head bone powder preparation

Fish bone powder was prepared as previously reported [16] with slight modification. The catfish heads were soaked in a hot (90 °C) alkaline solution (0.8 % NaOH, w/v) for 1 h at a ratio of bone-to-alkaline solution of 1:2 (w:v). Samples were rinsed with water until the pH of the water flowing through it reached approximately 7.0. The bone samples were autoclaved at 121 °C (350 g cm^−2^) for 1 h and dried overnight at 105 °C in an oven until constant weight. Dried samples were ground using a hammer mill (Retsch, Haan, Germany) and sieved to collect only bone particles that were smaller than 38 μm in diameter. This is referred to as catfish bone powder (CBP).

### 2.2 CBP characterization

#### 2.2.1 Chemical composition

Proximate composition of CBP was estimated according to AOAC standard methods [17]. The moisture content was determined by drying the powder in an oven at 105 °C for more than 12 h. A Soxhlet extraction was employed to estimate the crude fat content using petroleum ether as a solvent. The total nitrogen content was determined based on the Kjeldahl method to estimate the crude protein content using a 6.25 conversion factor [16]. The total mineral content was determined using a dry ashing method from the average values of at least two replicates.

#### 2.2.2 Total Ca and P contents

CBP was digested with 0.1 M nitric acid at 95 °C for 2 h before being atomized by inductively coupled plasma-optical emission spectrometry (ICP-OES, Model Optima 4300 DV, PerkinElmer Instruments, Norwalk, CT, USA). The emitted wavelengths for Ca and P were 317.933 and 213.617 nm, respectively, reported as mg/kg in the CBP.

#### 2.2.3 Determination of soluble Ca

CBP (1 g) was mixed with DI water (100 mL) prior to heating and shaking for 10 min. The mixture was passed through filter paper with the diameter of 11 μm (Whatman® No. 1). A filtrate was collected for Ca determination as described above. The amount of Ca in the filtrate was reported as soluble Ca.

#### 2.2.4 Determination of color values of CBP

Color values of CBP were evaluated using a colorimeter (CR-10; Minolta; Tokyo, Japan). The Hunter L*, a*, and b* values were reported from three replicates with at least five measurements.

#### 2.2.5 Determination of morphology of CBP

A field-emission scanning electron microscope (FE-SEM) equipped for energy dispersive spectroscopy (EDS) was used to analyze the morphology of CBP. The CBP was mounted prior to imaging and coated with gold for effective conductivity.

X-ray diffractometry (XRD) with a 2θ range of 10°–80° was applied to evaluate the crystallographic structure of CBP.

Fourier transform infrared spectroscopy (FT-IR) of CBP was done. The functional groups of samples were interpreted from FT-IR spectra, which were deconvoluted from wavenumbers in the range of 4000–400 cm^−1^.

### 2.3 Optimization of Ca extraction from CBP

Optimization of Ca extraction was designed using the Design Expert 7.0 software (Stat-Ease, Inc., Minneapolis, USA). Four effective parameters, microwave power (W), extraction time (s), CBP weight (g) and citric acid concentration (M) were selected as independent factors. For optimization, the highest, high, center, low and the lowest levels of each independent variable were coded as +α, +1, 0, −1 and -α, respectively (Table 1). The central composite design (CCD) experiments were used to evaluate the interactions of the independent parameters and optimize the levels of the process parameters. The experimental domain for independent parameters for the highest and the lowest was entered in terms of an alpha value of 2 to give 30 experiments with 16 factorial points, 8 axial points, and 6 central points [18]. According to the principles of CCD, 30 experimental sets were performed by manipulating the variable levels to identify the most favorable conditions for Ca extraction in the experimental design matrix (Table 1).

**Table 1:** Ranges of independent factors and their real and coded values.

Level of independent factor	Factor			
	A: citric acid (M)	B: time (s)	C: weight (g)	D: microwave power (W)
-α	0.05	30	0.10	100
−1	0.10	60	0.20	300
0	0.15	90	0.30	450
+1	0.20	120	0.40	600
+α	0.25	150	0.50	800

After extraction, the Ca content was determined using a titration method and reported as the response of the CCD. A quadratic model was evaluated using ANOVA along with other parameters such as the correlation coefficient (R^2^), adjusted R^2^ and predicted R^2^. The ideality for Ca extraction was optimized using a numerical desirability function [19]. Based on the four independent variables, 3D surface plots were obtained at five levels.

### 2.4 Quantification of Ca content extracted from CCD

The Ca content in the extracts was quantified using a titration method with slight modification [20]. CBP was digested in a citric acid solution (100 mL) under the conditions specified by the CCD. The pH of the digested sample was neutralized using NaOH prior mixing with an EDTA solution (0.01 M) and buffer (NH_4_OH–NH_4_Cl). The obtained solution was titrated with 0.01 M of a ZnCl_2_ standard solution using Eriochrome Black T as an indicator and expressed as mg Ca/mL.

### 2.5 Alginate bead formation induced by soluble Ca

Soluble Ca was extracted from CPB (0.4 g) at a microwave power of 300 W with a citric acid concentration of 0.1 M for 60 s. This Ca extract was used to induce gelation of a drop of an alginate solution (1.00 %). Using a burette, the alginate solution was dropped onto Ca extract to evaluate the potential of edible bead formation. The effect of Ca concentration on bead formation and hardness was tested at 3 different values, which are 1.13, 0.565, and 0.226 mg/mL. These values were obtained by diluting the extracted Ca solution for 2 and 5 folds. The formation of beads was checked. Only the formed beads (diameter ≈ 0.2 mm) were tested for hardness using a TA-XT2 instrument (Micro Stable Systems, Godalming, UK) in a puncture mode. The mean value of hardness was obtained from at least 10 measurements.

## 3 Results and discussion

### 3.1 Chemical properties of CBP

Proximate analysis of CBP clearly shows that CBP is a dried powder with a low moisture content (Table 2). Although the lipid content in fish varies from species to species, these compounds were found at high amounts in the bone, especially in the fish heads. Soaking fish heads in hot alkaline eliminated the lipids, resulting in a reduced crude fat content (3.51 %) which was lower than that presented in tilapia bone powder prepared from the main frame of the fish. Catfish is considered a high fat fish species but its bone powder could be stable with regard to oxidative rancidity due to its lower lipid content. The protein content in CPB was approximately 22 %. This value was higher than that of tilapia bone powder although it was still lower than in other fish species (26–41 %), *e.g.*, cod, saithe, blue whiting, salmon, trout, herring, and mackerel, among others [3]. The alkaline solution was not effective in ridding protein from bones since most of these proteins are stroma proteins, especially collagen. Stroma protein is resistant to both acid and alkaline solutions. The ash content was a main constituent in CPB, accounting for about 65 % of its mass. This value was lower than that of bone powder prepared from tilapia and silver carp [16,21]. Additionally, the ash contents in bone powder from several fish species are different and could be up to 40 %. Application of an alkaline solution was the most practical method to remove proteins, as indicated by the ratio of ash/protein. This value for CBP was 2.94 which is higher than that of salmon (1.00), but still lower than that found in tilapia bone powder (5.35). However, ash/protein is the criteria to indicate the effectiveness of protein removal. Although CPB has high protein content but it is still exhibiting a potential of mineral source due to high total ash content. The calcium (Ca) and phosphorous (P) are focused as the main elements found in bone materials and the ratio of Ca is higher than P for about 13 folds (Table 2). It is normally complex to each other in the crystal form of hydroxyapatite (HA) [3]. Therefore, low solubility of Ca was observed (Table 2). Extraction/digestion of this complex is required in order to fully utilization of Ca in fish bone powder.

**Table 2:** Chemical and physical properties of CBP.

Properties	Attribute	Content/Value
Chemical composition (%)	Moisture	1.83 ± 0.14
	Crude protein	22.96 ± 0.14
	Crude fat	3.51 ± 0.07
	Total ash	67.53 ± 0.02
	Total calcium	1.48 ± 0.00
	Total phosphorous	0.11 ± 0.00
Color Value	L*	88.92 ± 0.08
	a*	0.07 ± 0.02
	b*	8.40 ± 0.06
Solubility (%)		0.03 ± 0.0038

### 3.2 Color properties

CBP appears as a white powder as evidenced by high L* value (Table 2). However, its L* value was lower than that observed for tilapia bone powder [16]. The color of fish bone powder correlates well with the organic residues present in the powder. A high content of organic compounds results in a darker powder. Additionally, CBP has higher crude protein content and changes of aminos acids in the powder might be the main factor causing the lower L* value in CBP than observed for tilapia bone powder. Therefore, different source of fish bone and preparation method directly governed the color appearance of the fish bone powder. Application of this powder would be limited when changes of color were found during storage.

### 3.3 Morphology of CBP

#### 3.3.1 FE-SEM and EDS

**Fig. 1:**
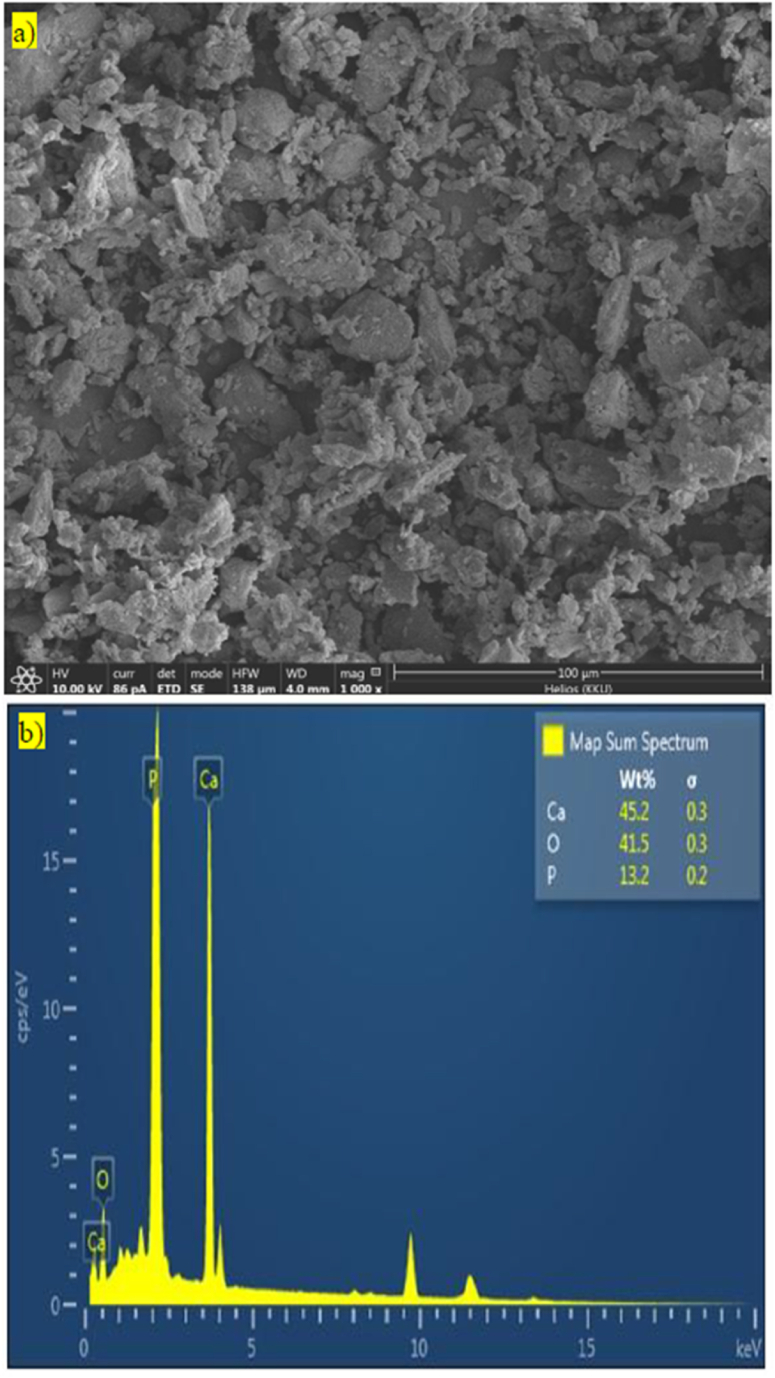
Images with 1000× magnification **(a)**, and elemental composition **(b)** of CBP under FE-SEM-EDS.

The FE-SEM image of CBP exhibited an asymmetric shape with little agglomerate and small pores. This might be due to dry milling process of hammer mill. In addition, sieving only particles less than 38 μm could selected microparticle with the average size of 9.15 ± 6.45 μm (Fig. 1a). Moreover, the elemental composition could be analyzed by FE-SEM-EDS mode. It is clearly confirmed that CPB is a good source of the Ca and P (Fig. 1b). This data was in agreement with the proximate analysis, which exhibited CBP is a good source of these elements. However, the arrangement in the bone matrix is needed to be analyzed.

#### 3.3.2 XRD analysis

**Fig. 2:**
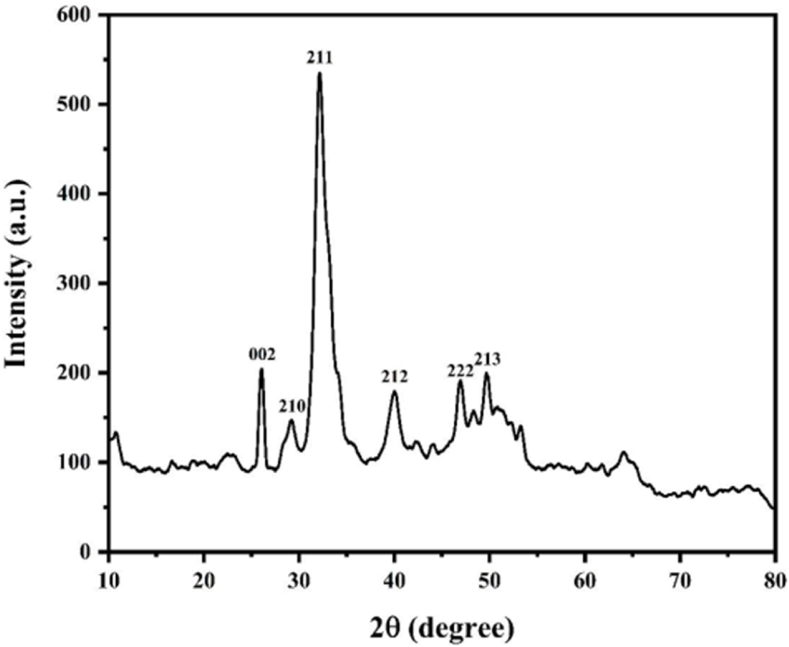
XRD diffractogram of CBP.

The XRD analysis of CBP clearly show the peaks related to HA crystals with diffraction lines at 2Ɵ angles of 26, 29, 32, 39, 47, and 49° (Fig. 2). These were corresponded to the (002), (210), (211), (212), (222), and (213), respectively. The broad spectrum of HA diffraction patterns exhibited a low crystallinity as reported previously [22]. The spectrum of the CBP sample is in good agreement with the reference model of phase-pure hydroxyapatite (JCPDS no. 09–0432). The similarity of the sample and standard HA diffraction patterns strongly indicates the presence of Ca-HA in the CBP sample. Although CBP is a good source of Ca, complexation in the form of HA is hardly solubilized. Therefore, extraction or digestion method would be applied in order to create the functional properties in food system.

#### 3.3.3 FT-IR spectra

**Fig. 3:**
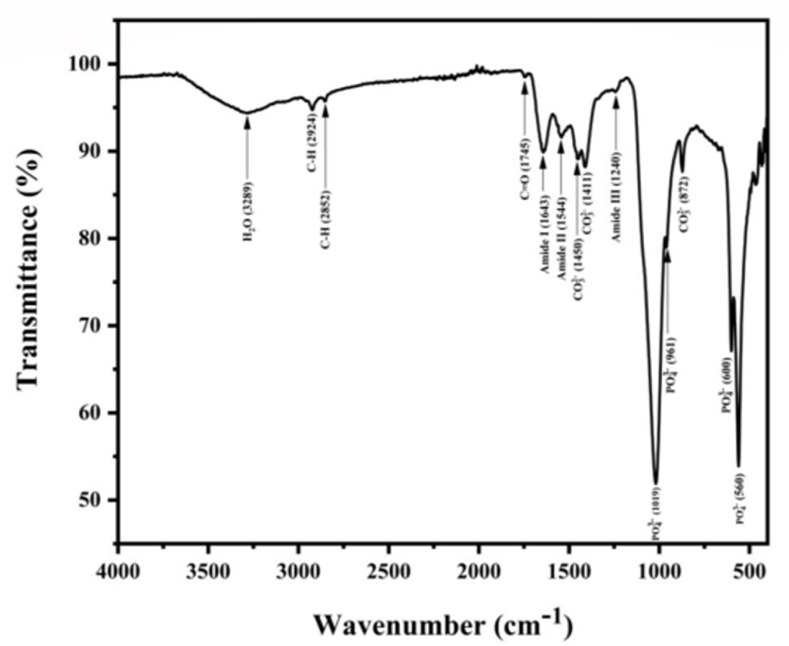
FT-IR spectrum of CBP.

The FT-IR spectrum of CBP showed prominent peaks at wavenumbers of 1019 and 550-600 cm^−1^ (Fig. 3), which are attributed to phosphate groups [[23], [24], [25]]. Moreover, absorption bands at wavenumbers of 560 and 600 cm^−1^ were form the presence of crystalline phosphate as Ca-HA. Peaks at 872, 1411 and 1450 cm^−1^ were assigned as a carbonate band [26]. Peak bands at 2852 and 2924 cm^−1^ indicated the presence of organic material in the sample [26] and the peak at 3289 cm^−1^ indicates water in the sample [27]. Those organic compound might be would be from proteins as indicated by proximate analysis. Moreover, the hypothesize was strongly confirmed by presenting of amide bands due to secondary structure of proteins. Those bands were at the peak 1,643, 1,544, and 1240 cm^−1^ assigned to be amide I, amide II, and amide III, respectively [28]. Those signals were attributed from the bending and stretching of secondary structure of collagen type II [[26], [28], [27]]. Based on the FT-IR spectra, CBP contain high Ca which is complexed with P in the HA complex as well as integrated with collagen matrix. Therefore, liberation of Ca from CBP requires hard conditions to digest collagen protein matrix and disintegrate the HA complex. Therefore, digestion of CBP under microwave using citric acid were studied. Optimization the process in order to obtain the optimal conditions based on the function of microwave power, digestion time, citric acid concentration, and CBP weight were evaluated as the same time using RSM methodology.

### 3.4 Optimization of Ca extraction

The traditional method of optimization does not show the combined effects of different input variables. So, the current study used RSM to optimize the variables and determine the combined effects of different input parameters (citric acid concentration, extraction time, weight, and microwave power). Experiments (30 sets) were conducted according to the CCD in order to determine not only optimum conditions for extraction of calcium but also the interactive effect of 4 factors. The experimental design for different variables with their coded and actual values are shown in Table 3. F-value and p-value, calculated from CCD model for calcium extraction are presented in Table 4. The F-value of 230.43 implies that the model is significant for calcium extraction. The large F-values of the model was due to low noise (<0.01 %). Based on this experiment, A, C, AC, A^2^, and B^2^ of the model were reported to be significant different (*p* < 0.05). The variance analysis suggested factor A (citric acid concentration) and C (CBP weight) affected significantly for Ca extraction. Moreover, the interaction between citric acid concentration and weight (AC) also played a crucial role in this process. The ‘lack of fit’, F-value (2.80) for Ca extraction was not significant.

**Table 3:** Experimental design in terms of actual factors of trial runs done for extraction of Ca using a citric acid solution.

Run	Independent variables				Ca content (mg/mL)	
	A Citric acid (M)	B Time (s)	C Weight (g)	D Microwave Power (W)	Actual	Predicted
1	0.20	120	0.20	600	0.49	0.52
2	0.20	60	0.20	600	0.51	0.50
3	0.20	120	0.40	600	1.04	1.05
4	0.15	30	0.30	450	0.78	0.78
5	0.10	120	0.20	300	0.54	0.54
6	0.20	120	0.20	300	0.50	0.50
7	0.10	60	0.40	300	1.14	1.13
8	0.15	90	0.10	450	0.28	0.28
9	0.05	90	0.30	450	0.85	0.87
10	0.20	60	0.20	300	0.47	0.50
11	0.15	90	0.30	450	0.85	0.84
12	0.15	90	0.30	450	0.85	0.84
13	0.15	90	0.30	800	0.81	0.81
14	0.15	90	0.30	450	0.85	0.84
15	0.10	120	0.20	600	0.56	0.56
16	0.10	120	0.40	300	1.12	1.13
17	0.20	120	0.40	300	1.00	1.03
18	0.15	150	0.30	450	0.83	0.81
19	0.15	90	0.50	450	1.38	1.36
20	0.15	90	0.30	450	0.81	0.84
21	0.10	60	0.20	300	0.58	0.57
22	0.20	60	0.40	600	0.98	0.99
23	0.15	90	0.30	100	0.83	0.81
24	0.20	60	0.40	300	1.00	1.00
25	0.25	90	0.30	450	0.76	0.71
26	0.15	90	0.30	450	0.84	0.84
27	0.10	60	0.20	600	0.56	0.56
28	0.10	60	0.40	600	1.11	1.11
29	0.15	90	0.30	450	0.84	0.84
30	0.10	120	0.40	600	1.15	1.14

**Table 4:** Analysis of variance (ANOVA) for calcium extraction.

Source	SS	df	MS	F-value	p-value	remark
Model	1.82	14	0.13	230.43	<0.0001	significant
A	0.038	1	0.038	66.91	<0.0001	
B	1.100E-003	1	1.100E-003	1.95	0.1832	
C	1.77	1	1.77	3132.77	<0.0001	
D	2.344E-006	1	2.344E-006	4.148E-003	0.9495	
AB	7.223E-004	1	7.223E-004	1.28	0.2759	
AC	3.235E-003	1	3.235E-003	5.73	0.0302	
AD	1.129E-004	1	1.129E-004	0.20	0.6613	
BC	1.016E-003	1	1.016E-003	1.80	0.1999	
BD	7.223E-004	1	7.223E-004	1.28	0.2759	
CD	1.914E-005	1	1.914E-005	0.034	0.8564	
A^2^	4.268E-003	1	4.268E-003	7.55	0.0149	
B^2^	3.851E-003	1	3.851E-003	6.82	0.0197	
C^2^	8.598E-004	1	8.598E-004	1.52	0.2363	
D^2^	1.799E-003	1	1.799E-003	3.18	0.0946	
Residual	8.474E-003	15	5.650E-004			
Lack of Fit	7.191E-003	10	7.191E-004	2.80	0.1335	not significant
Pure Error	1.283E-003	5	2.567E-004			
Cor Total	1.83	29				

SS: sum of squares; df: degree of freedom; MS: mean square; F: frequency; p: probability; Std. Dev.: Standard deviation; C.V. %: Coefficient of variance; PRESS: Predicted residual error sum of square; R^2^: Coefficient of determination; Adj R^2^: Adjusted – R^2^: Predicted – R^2^; Adeq Precision: Adequate precision; Std. Dev. = 0.024; Mean = 0.81; C.V. % = 2.93; PRESS = 0.043; R^2^ = 0.9954; Adj R^2^ = 0.9911; Pred R^2^ = 0.9764; Adeq Precision = 64.630.

**Fig. 4:**
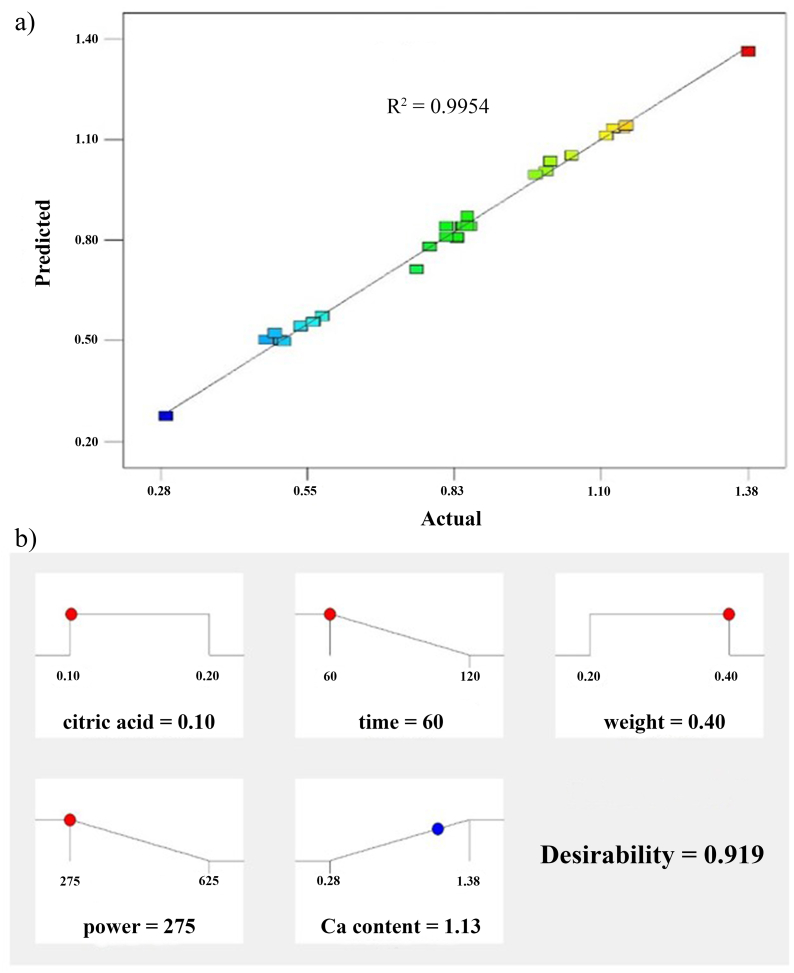
The parity plot exhibits the correlation between experimental and predicted values of Ca extraction **(a)**, and optimized ramps for the maximum Ca extraction obtained from CCD of RSM **(b)**.

The predicted R^2^ of 0.9764 is in reasonable agreement with the adjusted R^2^ of 0.9911. The signal to noise ratio was measured with adequate precision of 64.630, suggesting the precision of CCD/RSM. This was because the adequate precision is greater than 4. Thus, the R^2^ value at 0.9954 exhibited high correlation between the predicted and experimental data (Fig. 4a). This suggested that the actual values of Ca content from the experiments could be observed at the similar with the predicted values. The R^2^ is normally used to evaluate the confidence level and explained the suitability of the quadratic model. An optimized ramp (desirability) was used to predict optimal conditions for Ca extraction, which found to be at the citric acid concentration of 0.10 M, extraction time of 60 s, CBP weight of 0.40 g, and microwave power of 300 W. At this condition, Ca content of about 1.13 mg/mL would be obtained after extraction from CBP (Fig. 4b).

Interactions among the 4 independent variables and response (Ca content) is illustrated based on the regression model (Eq. (1)).

(1) Ca content = +0.84–0.040 * A + 6.771E-003 * B + 0.27 * C + 3.125E-004 * D + 6.719E-003 * A *B - 0.014 * A *C + 2.656E-003 * A *D + 7.969E-003 * B *C + 6.719E-003 * B *D −1.094E-003 * C *D - 0.012 * A^2^ - 0.012 * B^2^ - 5.599E-003 * C^2^ - 8.099E-003 * D^2^

**Fig. 5:**
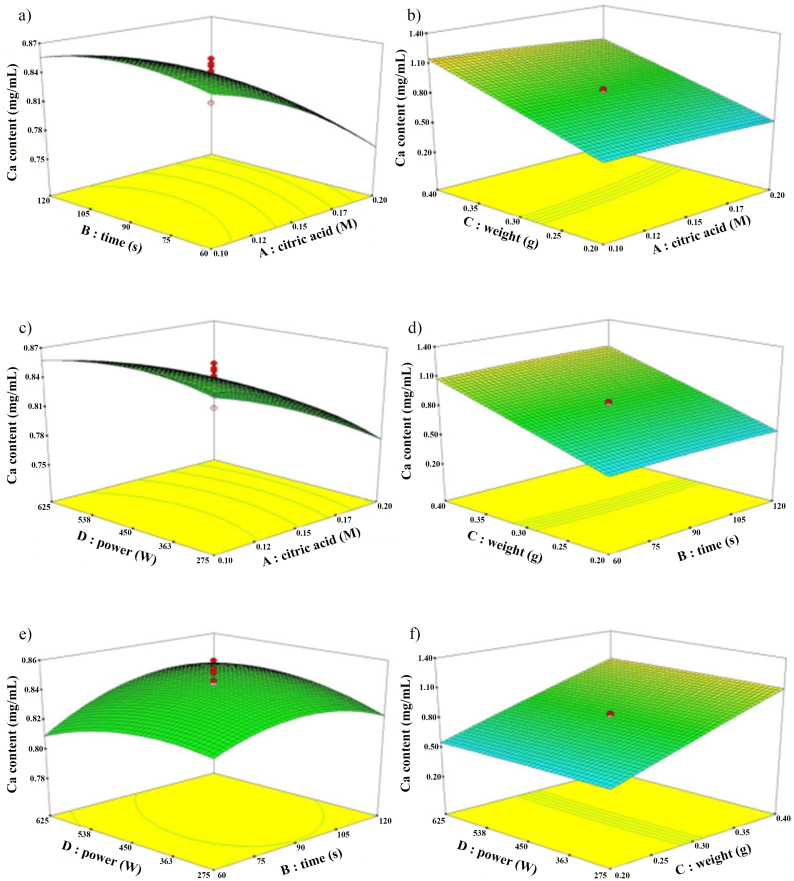
3D surface plots of Ca extraction from CBP based on the effect of citric acid concentration and extraction time **(a)**, citric acid concentration and CBP weight **(b)**, citric acid concentration and microwave power **(c)**, extraction time and CBP weight **(d)**, extraction time and microwave power **(e)**, and CBP weight and microwave power **(f)**.

The 3D surface plots of Ca content *versus* the citric acid concentration, time, weight, and microwave power are shown in Fig. 5. The Ca content changes slightly when extraction time increases (Fig. 5a). However, the Ca content seemed to decrease upon increasing the concentration of citric acid. This trend was also observed when effect of microwave powder and citric concentration were considered (Fig. 5c). A similar phenomenon was observed during extraction of Ca from the ashes containing hydroxyapatite using phosphoric acid [29]. It can be hypothesized that an increase in citric acid concentration may extract more Ca from CBP but that soluble Ca might be chelated by citrate, ionized from citric acid, to be a stable complex (calcium citrate). The previous report [30] suggested the higher chelating ability of citrate than EDTA toward the soluble Ca. This was evidenced by higher K_f_ between Ca and citric (K_f_ = 10^27.6^) than that value between Ca and EDTA (K_f_ = 10^10.7^). While quantification of Ca by back titration with EDTA, soluble Ca in the extract would likely more favorable to chelate with citrate in solvent rather than that with EDTA. Therefore, a reduction of soluble Ca up on increasing concentration of citric acid was observed. Similar results were obtained during extraction of an active calcium from *Megalobrama Amblycephala* bone using a mixed citric acid and malic acid by Lin et al. (2022). They demonstrated that extraction yield of active calcium decreased with increasing in the ratio of acid. Citric acid or citrate ion species formed stable complexes with calcium ions on the surface resulting in the dissolution of the bone powder sample [31]. This would be the limitation for using citric acid as the solvent during digestion of calcium from fish bone.

The effect of citric acid concentration and weight on Ca content was represented in Fig. 5B. The Ca content changed rarely with increasing in the citric acid concentration, while efficiency of Ca extraction was more relied on an increase of CBP weight. This result was agreed with that in Fig. 5d, which shows that CBP weight play more important role on Ca extraction than extraction time. Fig. 5e showed that the Ca content changed slightly with increasing in extraction time. The Ca content slightly increases initially and then decreases with increasing of microwave power. The Ca content changed rarely with increasing in microwave power was confirmed by the result in Fig. 5 (f). Based on the interactions among studied factors, there were 2 factors (citric acid concentration and CBP weigh) affecting the efficiency of calcium digestion/extraction under this system. Further study the effects of these two factors on calcium contents would let to more described the extraction efficiency.

In order to test the feasibility of the RSM, the verification experiment was set up at the optimal condition for Ca extraction (citric acid concentration: 0.10 M, extraction time:60s, CBP weigh: 0.40 g). However, microwave power was set up at 300 W instead of 275 W due to the limitation of heating scale designed by microwave specification. The actual Ca content in extract averaged from triplication was observed at 1.11 mg/mL. It can be clearly seen that the actual and predicted values of Ca content were found to be very close, providing such a low relative error at 1.77 %. Therefore, prediction model from RSM was promising technique feasible for application in Ca extraction from fish bone powder using citric acid. The RSM was also applied successfully to extract active calcium from *Megalobrama Amblycephala* and found the optimum extraction time for 0.8 h at 113 °C [32]. Our results provide more efficiency by shortening the extraction time to be 60 s due to application of microwave digestion.

It can be noted that our microwave oven has been modified by adapting the reflux condensation to reduce the evaporation of solvent during extraction. Thus, application of this optimal condition with the household microwave might yield slightly different results. Solvent evaporation could be observed, resulting in an increase citric acid concentration. Higher citric concentration could chelate the soluble calcium after extraction. This would be limited, while using the optimal condition obtained from this study.

### 3.5 Gelation of alginate induced by extracted Ca

Soluble Ca extracted at the optimal conditions was used to test the feasibility of its use as a functional food ingredient. Generally, Ca can induce gelation of a variety of polysaccharide solutions. A promising gelling agent is sodium alginate, SA (NaC_6_H_7_O_6_). It is the sodium salt of alginic acid, which is commercially extracted from seaweed, particularly *Macrocystis pyrifera* (kelp). It is a linear copolymer with homopolymer blocks of (1–4)-linked β-d-mannuronate (M) and α-l-guluronate (G) residues that can be induced to form hydrogels in the presence of appropriate ions, especially divalent cations such as soluble Ca [33]. The residual Ca (1.13 mg/mL) extracted from CBP under optimal conditions could induce gelation of a 1.0 % SA solution to form alginate beads. Formation of edible beads was still observed although the extract was diluted to half of its initial concentration (Ca≈ 0.565 mg/mL). However, the SA solution could not be gelled after diluting the Ca extract by 5-fold. This shows that gelation of an SA solution is dependent on Ca concentration. The hardness values of beads formed by the diluted Ca solutions was found at 98.37 ± 21.81 g force. However, this value increased (approximately 4 times) to be 405.87 ± 39.95 g force when double concentration Ca in the solution. This suggests that the hardness of bead is also dependent on Ca concentration. Therefore, soluble Ca extracted from CBP using citric acid acts as a cross-linker for hydrogel or hydro-bead formation**.** Formation of alginate beads induced by soluble Ca is already used commercially to process some imitation products, *e.g.*, caviar, salmon, and shrimp eggs [33]. However, using natural Ca to produce these products would favorably alter consumer perception. An edible bead production kit has been protected by plenty intellectual property of Thailand [34]. Additionally, natural Ca has been reported to be more compatible with biological functions [4]. Based on these results, extraction of natural Ca from CBP is a possible technological development to create innovative food products.

## 4 Conclusions

Bone powder from catfish heads is a good source of natural minerals, especially calcium and phosphorous. However, these elements are in a complex composite, hydroxyapatite, giving them lower solubility. Extraction of calcium from catfish head bone powder by citric acid using a microwave-assisted method was successfully optimized using RSM. The maximal calcium content can be extracted from fish bone powder (0.4 g) at microwave power of 300 W using citric acid concentration of 0.1 M for 60 s. The extracted calcium was sufficient to induce gelation of the sodium alginate drop. This bead forming ability and bead hardness were dependent on the calcium concentration. Calcium extraction from the bones of catfish heads is an alternative strategy to utilize fish by-products as a source of natural calcium to create innovative food products such as edible bead kit.

## Ethical statement

The data and experiments in this study comply with ethical policies.

## CRediT authorship contribution statement

**Manop Sriuttha:** Validation, Methodology, Investigation, Data curation. **Nachayut Chanshotikul:** Methodology, Investigation. **B. Hemung:** Writing – original draft, Funding acquisition, Conceptualization.

## Declaration of competing interest

All the authors declared that they have no financial/commercial conflicts of interest.
